# Disease-specific IgG Fc N-glycosylation as personalized biomarkers to differentiate gastric cancer from benign gastric diseases

**DOI:** 10.1038/srep25957

**Published:** 2016-05-13

**Authors:** Dan Zhang, Bingchao Chen, Yanmin Wang, Peng Xia, Chengyan He, Yujie Liu, Ruiqing Zhang, Mo Zhang, Zhili Li

**Affiliations:** 1 Department of Biophysics and Structural Biology, Institute of Basic Medical Sciences, Chinese Academy of Medical Sciences and School of Basic Medicine, Peking Union Medical College, Beijing 100005, P.R. China; 2 Department of Clinical Laboratory, Heze Municipal Hospital, Shandong 274031, P.R. China; 3 Department of Oncology Surgery, First Affiliated Hospital, Xi’an Jiaotong University, Xi’an, 710061, P.R. China; 4 Laboratory Medicine Center, China-Japan Union Hospital of Jilin University, Changchun, 130041, P.R. China

## Abstract

Interest in the pathophysiological role of IgG fragment crystallizable (Fc) N-linked glycosylation arose from changes in humoral immune responses. In this study, circulating disease-specific IgG (DSIgG) derived from serum immunoinflammation-related protein complexes was isolated from 846 serum samples of 443 patients with benign gastric diseases (BGDs) and 403 patients with gastric cancer (GC), and DSIgG glycopeptides attached to IgG Fc region at the site of Asn297 were analyzed using matrix-assisted laser desorption/ionization- Fourier transform ion cyclotron resonance mass spectrometry (MALDI-FTICR MS). A total of 22 glycopeptides were detected. Statistical analysis indicated that DSIgG1 G1S, DSIgG2 G0F, G1, G2F, and G2FS as well as DSIgG2 galactosylation and sialylation are significantly associated with sex in BGD patients and that the age-specific glycoforms and glycosylation features from DSIgG between BGD patients and GC patients have similar change trends. In addition, significant changes in galactosylation, sialylation, and bisecting N-acetylglucosamine (GlcNAc) from DSIgG were also observed between two pathophysiological states. Receiver operating characteristic (ROC) analysis indicated that the G2FN/G1FN (from DSIgG2) ratio has an excellent capability to distinguish female BGD patients from female GC patients over the age range of 20–79 years, with the sensitivity of 82.6%, the specificity of 82.6%, and the area under curve (AUC) of 0.872.

Gastric cancer (GC) is one of the leading cause of cancer-related death worldwide, contributing to 8.8% of the cancer mortality^1^. Globally, 0.7 million patients with GC die annually, making GC a highly lethal malignancy^1^. GC has large geographical differences in cancer mortality, incidence, and prevalence. Specifically, it is one of the most prevalent cancer in Eastern Asia, such as China^2^. Furthermore, GC incidence in males is twice as high as that in females^3^. At present, GC diagnosis mostly relies on endoscopy, and partly relies on symptoms reported by patients in western countries^4^.

Glycosylation is one of the most important post-translation modifications of proteins. Changes in glycosylation can significantly modulate the structure and function of glycoproteins^5^, and altered glycoforms are associated with several physiological and pathological states, along with pathogenesis and progression of cancer^6^,^7^,^8^. It is found that currently used cancer biomarkers, such as carbohydrate antigen(CA)15-3, CA19-9, CA125, and carcino-embryonic antigen (CEA), are glycoproteins^9^, and their glycosylation has attracted wide attention^10^. For past decades, the majority of studies have focused on glycosylation analysis of global serum glycoproteins, especially for glycans released from total serum glycoproteins^11^,^12^. Recently, several studies have paid more attention on glycosylation analysis of individual proteins^13^,^14^,^15^,^16^, especially for immunoglobulin G(IgG)^17^,^18^,^19^.

IgG is the most abundant serum glycoprotein that plays a key role in adaptive immune response. IgG can trigger antibody-dependent cell-mediated cytotoxicity (ADCC) or complement-dependent cytotoxicity (CDC) through the interaction between its fragment crystallizable (Fc) region and Fcγ receptors of innate immune effector cells or complement components to eliminate non-self invaders and abnormal cells such as cancer cells^20^. This interaction can mediate pro- and anti-inflammatory activities via the Fc N-linked glycans attached at the site of Asn297. Previous studies have indicated that changes in Fc N-glycosylation partly reflect human health states^21^,^22^,^23^,^24^. Total serum IgG is usually obtained using Protein A and Protein G, and large amounts of steady-state IgG also exist in healthy state, which are non-functional and merely mediate clinical protection^25^. So far, the isolation of disease-specific IgG (DSIgG) still remains a challenge.

Our previous studies have found that serum immunoinflammation-related protein complexes (IIRPCs) are closely associated with disease states, disease types, and the progression of lung cancer^26^,^27^,^28^. Their major components are complements, haptoglobin, immunoglobulin A, and IgG. In this study, we employed a combined approach of an optimized native polyacrylamide gel electrophoresis (native-PAGE) and sodium dodecylsulfonate-PAGE (SDS-PAGE) to isolate DSIgG, followed by the detection of the glycopeptides derived from DSIgG using matrix-assisted laser desorption ionization-Fourier transform ion cyclotron resonance mass spectrometry (MALDI-FTICR MS). Finally, changes in the levels of DSIgG glycoforms between benign gastric diseases (BGDs) and GC were statistically analyzed, and receiver operating characteristic (ROC) analysis indicated that glycoform ratio (G2FN/G1FN from DSIgG2) has a powerful capability to distinguish female BGD patients from female GC patients over the age range of 20–79 years, with the sensitivity of 82.6%, the specificity of 82.6%, and the area under curve (AUC) of 0.872.

## Results

### N-glycopeptide profiling of DSIgG

**Figure 1:**
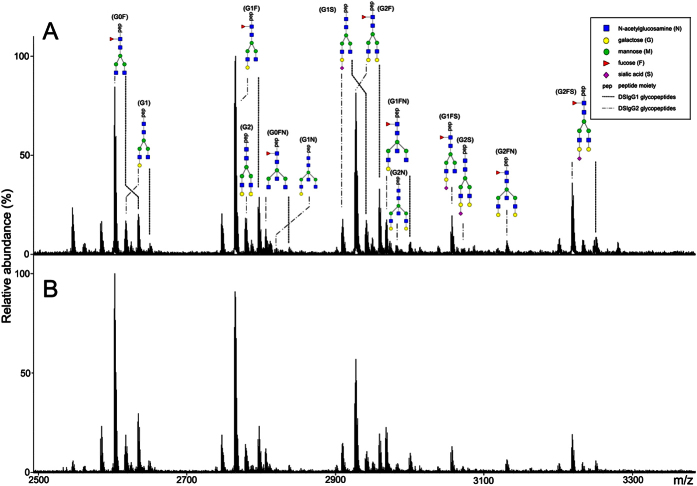
Representative mass spectra of the glycopeptides from tryptic digests of DSIgG from one BGD patient (**A**) and one GC patient (**B**). 8 detected glycoforms (G0F, G0FN, G1, G1F, G1FN, G1S, G2F, and G2FS) were from DSIgG1 and 14 detected glycoforms (G0F, G0FN, G1, G1N, G1F, G1FN, G1S, G1FS, G2, G2S, G2N, G2F, G2FN, and G2FS) from DSIgG2. G0FN and G2FS from DSIgG1 and G2S from DSIgG2 undetected in one-third serum samples were excluded in further statistical analysis.

A total of 1037 serum samples from 525 patients with BGDs and 512 patients with GC were collected in this study. Serum IIRPCs, which are found to be positively correlated with pathophysiological states, were isolated using the native-PAGE and the results are shown in Supplementary information Fig. S1. MALDI-FTICR MS was employed for N-linked glycopeptides profiling of DSIgG. Representative mass spectra of the glycoforms derived from DSIgG are shown in Fig. 1, and the corresponding *m/z* values of the detected glycopeptides of DSIgG and their individual peptide sequences are listed in Supplementary Information Table S1. During the entire experiment, a quality control (QC) serum sample was used as external reference, and the QC sample was analyzed once every nine test serum samples. Intraday and interday precision were used to evaluate the reproducibility of the experiment. Relative standard deviations (RSDs) of the glycoforms distributed in almost equal interval of *m/z* value in mass spectra with middle intensity (*i.e.*, G0F at *m/z* 2602.0561, G1F at *m/z* 2764.1089, G0FN at *m/z* 2805.1355, G2F at *m/z* 2926.1617, G1FN at *m/z* 2967.1883, and G1FS at *m/z* 3055.2043) from DSIgG were calculated to evaluate the experimental precision during the whole experiment. It is found that the RSDs of 6 above-mentioned representative glycoforms from the QC sample were less than 20%, and the intraday and interday precisions were also less than 20%, which are acceptable for complex biological sample analysis.

In this study, nineteen glycoforms from DSIgG, six glycoforms (i.e., G0F, G1, G1F, G1S, G2F, and G1FN) from DSIgG1 and thirteen glycoforms (i.e., G0F, G1, G1F, G1N, G2, G0FN, G1S, G2F, G1FN, G2N, G1FS, G2FN, and G2FS) from DSIgG2 were extracted from the mass spectra of 846 serum samples (Supplementary Information Table S2). As 3- and 6-arm galactosylation could not be separated by MALDI-MS, the DSIgG1 glycoform in our study is the sum of the two forms. It should be noted that the intensities of missing glycoforms in mass spectra of certain serum samples were replaced by Expectation-maximization algorithm in SPSS for further statistical analysis^29^. To evaluate a potential effects of regional diversity on glycoforms, partial least squares-discriminate analysis(PLS-DA) was performed. The statistical results show that there is no association between region and the detected glycoforms, with the predicted residual sum of square (PRESS) of 0.9667 for BGD patients and 0.8635 for GC patients (Supplementary information Fig. S2).

### Sex-specific DSIgG Fc N-glycosylation

**Figure 2:**
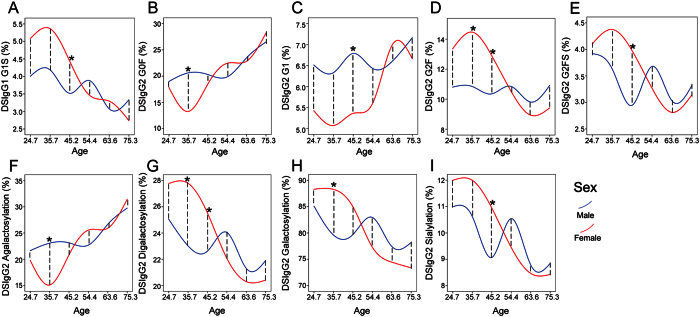
Change trends of sex-specific glycoforms and glycosylation features derived from DSIgG in six different age groups of BGD patients. The significance of sex-specific glycoforms from DSIgG1 (**A**) and DSIgG2 (**B**–**E**), along with glycosylation features from DSIgG2 (**F**–**I**) was calculated by Mann-Whitney U test followed by false discovery rate (FDR) controlling procedures. The scale of the x-axis represents the mean age of each age group for males and females. The curves are the interpolation lines of the mean intensities of each glycoform or glycosylation feature from males (blue) or females (red) in six different age groups. *P_*adj*_ < 0.05.

The correlation analysis between sex and the glycoforms was performed using Mann-Whitney U test in six different age groups (*i.e*., <30 years old, 30~39 years old, 40~49 years old, 50~59 years old, 60~69 years old, and >69 years old) for BGD patients and GC patients, respectively. And then false discovery rate (FDR) controlling procedures were used to obtain the adjusted *p*_*adj*_ values. It is worth noting that the glycoforms (*i.e.*, G1S from DSIgG1 and G0F, G1, G2F, and G2FS from DSIgG2 in Fig. 2A–E) and glycosylation features (*i.e.*, agalactosylation, digalactosylation, galactosylation, and sialylation from DSIgG2 in Fig. 2F–I) in BGD patients have exhibited a strong sex correlation(*p*_*adj*_ < 0.05), while for GC patients, no sex correlations was observed.

### Age-specific DSIgG Fc N-glycosylation

**Figure 3:**
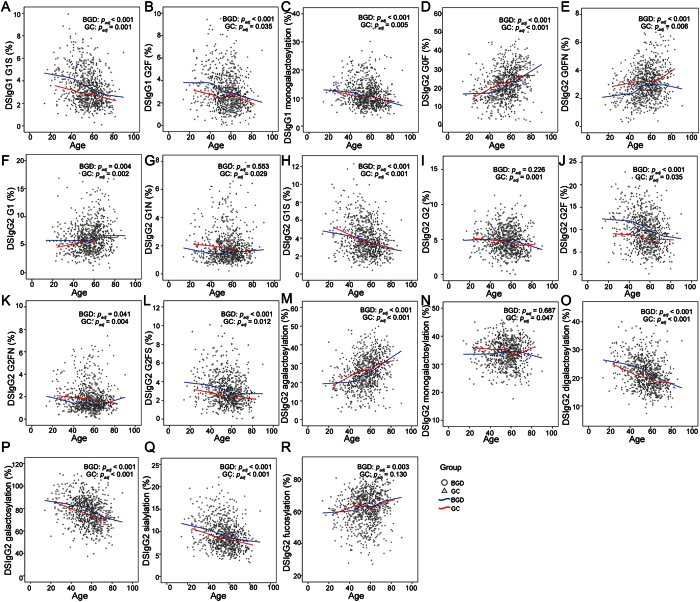
Change trends of age-specific glycoforms and glycosylation features derived from DSIgG. The significances of the age-specific glycoforms and glycosylation features from DSIgG1 (**A**–**C**) and DSIgG2 (**D–R**) was calculated using Spearman correlation analysis followed by false discovery rate (FDR) controlling procedures. The curves are the fit lines of glycoforms or glycosylation features from BGD patients (blue) or GC patients (red). A *p*_*adj*_ less than 0.05 represents statistical significance.

Based on the sex-matched BGD patients and no sex-correlated GC patients, age-specific DSIgG Fc N-glycosylation analysis could be performed based on the participants listed in Supplementary Information Table S2. Correlation analysis between age and glycoforms was performed using Spearman correlation analysis followed by FDR controlling produces in BGD patients and GC patients, respectively. The results indicated that 12 glycoforms (i.e., G1S and G2F from DSIgG1 and G0F, G0FN, G1, G1N, G1S, G2, G2F,G2FN, and G2FS from DSIgG2) are age-specific. As shown in Fig. 3, these glycoforms show a similar change trend in both BGD patients and GC patients, except for G1N and G2 of DSIgG2 for BGD patients (Fig. 3G,I). It should be noted that with increased age, the galactosylation and sialylation of DSIgG2 in both BGD patients and GC patients were significantly decreased (Fig. 3P,Q) and for elderly patients, the monogalactosylation (G1 + G1F + G1S + G1FN) of DSIgG1 was also decreased in BGD and GC patients (Fig. 3C), while the fucosylation of DSIgG2 was significantly increased in BGD patients (Fig. 3R). Taken together, our findings indicate that sex and age were closely correlated with DSIgG Fc glycosylation.

### Correlation among glycoforms of DSIgG

To evaluate the correlations among different glycoforms derived from DSIgG, the correlation analysis was performed. The glycoform relationships between two different pathophysiological states have shown different behaviors (Supplementary information Fig. S3). Strong positive correlations between DSIgG1glycoforms (i.e., G1FN, G1S, and G2F) and DSIgG2 glycoforms (i.e., G2N, G2FN, and G1FS) were observed in both GC patients and BGD patients, while positive correlations between the DSIgG1glycoforms (i.e., G1FN, G1S, and G2F) and DSIgG2 glycoforms (i.e., G1N, G1S, and G2FS) were only observed in GC patients. The opposite correlations between the DSIgG1 glycoforms (i.e., G1, G1F, G1FN, G1S, or G2F) and DSIgG2 G0F in BGD patients and GC patients were observed, positive for BGD and negative for GC. In addition, the correlation analysis of glycosylation features indicated that a negative correlation between the fucosylation and the sialylation as well as bisecting N-acetylglucosamine (GlcNAc) of DSIgG2 was observed in GC patients instead of BGD patients.

### Change trends in DSIgG glycosylation between GC patients and BGD patients

Age- and sex-matched participants are randomly divided into training set and validation set. Characteristics of participants in the two sets are listed in Table 1. Mann-Whitney U test was used to screen differential DSIgG glycoforms, glycoform ratios, and glycosylation features between BGD patients and GC patients, followed by FDR Controlling Procedures to obtain the adjusted *p*_*adj*_ values. For the training set, the analytical results are listed in Table 2. As shown in Table 2, the levels of DSIgG2 glycoforms (i.e., G0F, G0FN, G1N, and G2FN) and DSIgG1 glycoforms (i.e., G1 and G1FN) in GC patients were significantly increased, while the levels of DSIgG2 glycoforms (i.e., G2, G2F, G1FS, and G2FS) and DSIgG1 glycoforms (i.e., G1S and G2F) in GC patients were remarkably decreased compared with BGD patients. Glycoform ratios derived from DSIgG2 (i.e., G2FN/G1FN, G1N/G1, G2N/G2, G2FN/G2F, and G2FN/G2N) and G1FN/G1F from DSIgG1in GC patients were significantly larger than those of BGD patients, while the glycoform ratios of DSIgG2 (i.e., G2F/G1F, G2F/G0F, G1FN/G0FN, and G1FN/G1N) and of DSIgG1 (i.e., G2F/G1F, G2F/G0F, G1F/G1, and G1S/G1) in GC patients were significantly decreased compared with BGD patients. Statistical analysis of glycoform features indicated that digalactosylation, galactosylation, and sialylation of DSIgG2 in GC patients were markedly decreased while bisecting GlcNAc of DSIgG2 in GC patients was significantly increased compared with BGD patients.

To evaluate the above-mentioned results, the variables, which have statistical significant between BGD and GC patients (*p*_*adj*_ < 0.05), have further been confirmed based on an independent validation study. The statistical analysis showed that the above-mentioned variables have similar change trends in the validation set except DSIgG2 G2 (Table 2).

**Figure 4:**
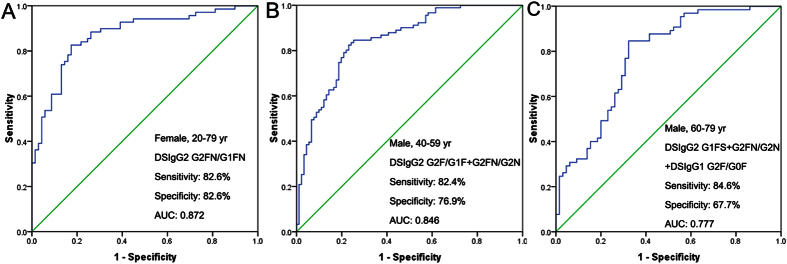
ROC curves. ROC analysis of the validation set to distinguish female BGD patients from female GC patients over the age range of 20–79 years (A, 138 cases); to distinguish male BGD patients from male GC patients over the age range of 40–59 years (B, 182 cases), and to distinguish male BGD patients from male GC patients over the age range of 60–79 years (C, 130 cases).

ROC analysis of the independent validation set indicated that G2FN/G1FN ratio derived from DSIgG2 for females aged from 20 to 79 years old has a excellent capability to differentiate GC patients from BGD patients, with the sensitivity of 82.6%, the specificity of 82.6%, and the AUC value of 0.872 (Fig. 4A). For males aged from 40 to 59 years old, ROC analysis indicated that a combination of G2FN/G2N and G2F/G1F from DSIgG2 has provided a powerful ability to differentiate GC patients from BGD patients, with the sensitivity of 82.4%, the specificity of 76.9%, and the AUC of 0.846 (Fig. 4B), and for males aged over 60 years old, the panel of DSIgG2 G1FS, DSIgG2 G2FN/G2N ratio, and DSIgG1 G2F/G0F ratio has a good ability to differentiate GC patients from BGD patients, with the sensitivity of 84.6%, the specificity of 67.7%, and the AUC value of 0.777 (Fig. 4C).

## Discussion

Recently, emerging evidence indicates that changes in the glycosylation of total serum IgG isolated using Protein A or Protein G are associated with pathophysiological states^30^,^31^,^32^,^33^,^34^,^35^. In this study, we employ our developed approach to obtain DSIgG, which only exists in patients with chronic diseases^26^. Mass spectrometric analysis indicated that DSIgG is mainly composed of two subclasses of IgG1 and IgG2. Statistical analysis indicated that DSIgG glycoforms are closely correlated with sex, age, and pathophysiological states, which is in good agreement with previous studies^32^,^36^. It should be noted that the sex-specific DSIgG glycoforms with one or two terminal galactose residue(s) were observed in BGD patients and that the age-specific DSIgG glycoforms have similar change trends in both BGD patients and GC patients, except for DSIgG2 G1N, DSIgG2 G2, and DSIgG2 monogalactosylation in BGD patients. Previous studies have indicated that alteration of IgG glycoforms with age is correlated with change in age-specific B-cells^37^,^38^ and that changed hormones may be responsible for sex-specific glycosylation changes^32^. The strong negative correlation between DSIgG1 monogalactosylation and DSIgG2 fucosylation is significant in both BGD and GC patients (*p*_adj_ > 0.001), which may be due to the association between the biological functions and the effector properties of IgG subclasses^39^, along with the same series of glycosyltransferases^40^. In addition, the correlation analysis of DSIgG glycoforms indicated that DSIgG glycosylation has different behaviors between GC and BGD patients, which may be ascribed to the production of tumor-associated autoantibody^41^. Taken together, our results indicate that BGD and GC patients may have different mechanisms of humoral immune responses.

Previous studies have shown that decreased galactosylation of total serum IgG was observed in rheumatoid arthritis, Crohn’s disease, and cancers compared with healthy individuals^32^,^33^,^34^,^35^,^42^,^43^,^44^. However, no study has been performed to compare the difference in the glycoforms of DSIgG between BGD and GC patients. In our study, significantly decreased galactosylation and sialylation of DSIgG in GC patients were observed compared with BGD patients, which is consistent with previous study on the glycosylation of total serum IgG in GC patients^35^. Galactosylation of IgG Fc portion can promote the association between IgG and Fcγ inhibitory receptors, resulting in an increase of IgG anti-inflammatory properties, while agalactosylation of IgG, exposing GlcNAc residues, increases the binding with mannose-binding lectin, resulting in promotion of CDC activity^42^,^45^. Sialylation of IgG Fc portion can increase anti-inflammatory properties of IgG^46^,^47^, and bisecting GlcNAc also plays an essential role in enhancing activity of IgG via increased interaction between IgG Fc portion and Fcγ activating receptors, resulting in pro-inflammatory responses^48^. In our study, decreased sialylation of DSIgG (i.e., DSIgG2 G1FS and G2FS and DSIgG1 G1S) in GC patients was detected compared with BGD patients, indicating that anti-inflammatory role of the sialylation of DSIgG might have different mechanisms or different grade inflammation between GC patients and BGD patients. It should be noted that significantly increased bisecting GlcNAc (i.e.,G0FN, G1N, and G2FN from DSIgG2 and DSIgG1 G1FN) in GC patients was observed compared with BGD patients, showing that bisecting GlcNAc may be associated with pro-inflammatory response. It was found that afucosylation of IgG enhances binding activities to Fcγ activating receptors, resulting in profoundly enhancing ADCC activity^49^, and increased fucosylation of IgG is associated with fetoneonatal alloimmune thrombocytopenia^50^. However, statistical analysis of fucosylated DSIgG glycoforms (G2F and G1FN from DSIgG1 and G0F, G0FN, G2F, G1FS, G2FN, and G2FS from DSIgG2) have not shown similar behaviors (Table 2), suggesting that structures and components (e.g., presence or absence of G and/or S) of glycans as well as peptide backbone attached to glycans may influence their pro- or anti-inflammatory roles.

Significantly increased DSIgG1 glycoforms (i.e., G1 and G1FN) and DSIgG 2 glycoforms (i.e., G1N and G2FN) in GC patients relative to BGD patients suggests that these glycoforms with one or two terminal galactose residues have pro-inflammatory role, while remarkably decreased G1S and G2F from DSIgG1 and G2, G2F, G1FS, and G2FS from DSIgG2 in GC patients compared with BGD patients implies that these glycoforms with one or two terminal galactose residues have anti-inflammatory role. These findings further indicate that, for complex-type glycoforms, synergistic action of galactosylation, sialylation, fucosylation, and bisecting GlcNAc residue as well as the peptide backbone attached to glycans may influence pro- or anti-inflammatory roles of individual glycoforms. Taken together, our results indicate that changes in DSIgG glycoforms may be personalized biomarkers to differentiate different pathophysiological states and that the diagnosis between GC/BGD indeed benefit from MALDI-MS analysis of tryptic glycopeptides originating from immunoinflammation-related protein complexes based on the process established in this study. ROC analysis have shown that G2FN/G1FN ratio of IgG2 has an excellent capability to differentiate female GC patients from female BGD patients, with the sensitivity of 82.6%, and the specificity of 82.6%, and the AUC value of 0.872 over the age range of 20 to 79 years. However, for males, changes in DSIgG glycoforms are closely associated with age. ROC analysis showed that DSIgG2 G2F/G1F  + G2FN/G2N has good ability to differentiate male GC patients from male BGD patients, with the sensitivity of 82.4%, the specificity of 76.9%, and the AUC value of 0.846 over the age range of 40 to 59, and that DSIgG2 G1FS + G2FN/G2N + DSIgG1 G2F/G0F shows a good ability to distinguish male GC patients from male BGD patients, with the sensitivity of 84.6%, the specificity of 67.7%, and the AUC value of 0.777 over the age range of 60 to 79.

There are some limitations in our study. First, for males, we did not recruit enough number of male patients with the age range of 20 to 40 years. Second, due the low amount of IgG3 and IgG4 relative to IgG1 and IgG2, we did not consider the glycoform contribution of DSIgG3 and DSIgG4 which may have the same *m/z* values of DSIgG1 and DSIgG2.

## Conclusions

In this study, we have applied a new strategy to isolate DSIgG from serum. Changes in DSIgG Fc glycoforms could reflect difference in pathophysiological states between GC and BGD. Our findings also indicate that humoral immune responses between GC and BGD may have different mechanisms. The synergistic effect of galactosylation, fucosylation, sialylation, and bisecting GlcNAc of DSIgG glycoform on pro- or anti-inflammatory role of DSIgG may plays essential roles in GC and BGD. More importantly, some glycoform features have shown excellent personalized diagnostic capability to differentiate GC from BGD.

## Method

### Serum sample collection

A total of 1037 serum samples from 525 BGD patients and 512 GC patients were collected from four regions (Beijing, Shandong, Shaanxi, and Jilin) of China, with 62% males and 38% females. Age of these participants is from 20 to 90 years old. All specimens were the remaining sera after clinical experimental examination. These serum samples were stored at −80 °C within 6 h of blood collection. This study was approved by the ethics committees of the Institute of Basic Medical Sciences, Chinese Academy of Medical Sciences & Peking Union Medical College, and informed consents were acquired from all patients. All experiments were performed in accordance with relevant guidelines and regulations.

### Study Design

1037 serum samples collected in this study include 512 GC patients and 525 BGD patients. 2 GC patients and 13 BGD patients without the information of sex or age were excluded before the native-PAGE separation. The remaining serum samples from 510 GC patients and 512 BGD patients were separated using an optimized native-PAGE as previously described^26^. It was found that 107 GC patients and 69 BGD patients had no IIRPCs and excluded in the following study^26^. Finally, 403 GC serum samples and 443 BGD serum were selected for the further experiments. Based on correlation analysis of DSIgG glycoforms, the age- and sex-matched samples were randomly classified into training set and validation set for biomarker discovery. The former includes 60 GC patients and 60 BGD patients and the latter is composed of 231 GC patients and 231 BGD patients.

### Isolation of serum IIRPCs

Native-PAGE was performed on the basis of the approach described previously with slight modifications^26^. Briefly, 5 μL serum was separated with the electrophoresis system consisted of separating gel (4% to 10% gradient acrylamide gel) and stacking gel (4%) to obtain serum IIRPCs. 9 serum samples and one QC serum sample were separately loaded into each lane in one native-PAGE gel. Electrophoresis was run at 10 mA per gel for 1.5 h, followed by 25 mA per gel for 3 h. The gels were stained overnight with Coomassie brilliant blue G-250 followed by destaining with water for at least 24 h. A commercially available native protein mixture (66–669 kDa) (GE Healthcare, Uppsala, Sweden) was used as molecular weight markers.

### DSIgG separation from serum IIRPCs

The above-mentioned gel bands (bands a1, a2, a3, and a4; bands b1, b2, b3, b4, and b5 in Supplementary Information Fig. S1) were excised and washed with ultrapure water. Then serum IIRPCs were in-gel denatured and reduced with 300 μL 0.2 M dithiothreitol for 45 min at 37 °C, followed by alkylation with 300 μL 0.5 M iodoacetamide for 30 min. After washed with ultrapure water, the gel band was ready for SDS-PAGE separation. Electrophoresis was performed at 60 V for 45 min, followed by 120 V for 2 h. Then the gels were stained with Coomassie brilliant blue G-250.

### Tryptic Digestion and Glycopeptide Enrichment

All DSIgG bands from one patient in SDS-PAGE (For pattern a, combined the bands a1, a2, a3, and a4 together for analysis. For pattern b, combined the bands b1, b2, b3, b4, and b5 together for analysis in Supplementary Information Fig. S4) were put together and cut into pieces. 50% acetonitrile (ACN) in 25 mM ammonium bicarbonate was used for destaining, and then 100% ACN for dehydration. 10 μL of 12.5 ng/μL trypsin (sequencing grade modified, Roche Diagnostics, Mannheim, Germany) in 25 mM ammonium bicarbonate was added to gel pieces and incubated at 37 °C for 16 h. The supernatant was collected and concentrated using a SpeedVacuum concentrator. Then the glycopeptides derived from DSIgG were enriched using the popcat-Tip that was made by packing poplar catkin from *Populus tomentosa* Carr. into a pipette tip. Briefly, the popcat-Tip was activated successively by 50 μL ultrapure water, 100% ACN, and 80% ACN. Tryptic digests were dissolved by 80% ACN, and then loaded into the popcat-Tip. After centrifuged at 1500 g for 5 min, the solution was removed, and then 30 μL of ultrapure water was added. The enriched glycopeptides from DSIgG were recovered after centrifuged at 1500 g for 5 min and concentrated using a SpeedVacuum Concentrator.

### Mass spectrometry profiling

All experiments were performed using 9.4 T Apex-ultra™ hybrid Qh-FTICR mass spectrometer (Bruker Daltonics, Billerica, MA,USA) equipped with a 200 Hz, 355 nm Nd:YAG laser. All spectra were acquired using ApexControl 3.0.0 (Bruker Daltonics). The eight peptides mixture (*m/z* 775.4137, *m/z* 913.4728, *m/z* 1046.5418, *m/z* 1296.6848, *m/z* 1619.8223, *m/z* 1936.8550, *m/z* 2465.1983, *m/z* 3147.4710) was used to calibrate the instrument over the m/z range of 500–4000 in positive ion mode at the resolution of 160,000 at m/z 400. The dried enriched glycopeptides were re-suspended in 2 μL ultrapure water. 0.3 μL of the supernatant was spotted onto a MTP AnchorChip™ plate and dried at room temperature, and then the sample spot was overlaid by 0.3 μL of matrix solution of 20 mg/mL 2, 5-dihydroxybenzoic acid in 50% ACN with 0.1% trifluoroacetic acid and allowed to dry at room temperature. Laser shots for each scan was 100 and approximately 30 scans were accumulated until the absolute intensity of the base peak reached to 5 × 10^7^.

### Data Analysis

The intensities of glycopeptides were generated by Data Analysis 4.0 software (Bruker Daltonics). First, known glycopeptide masses were used for internal calibration and the monoisotopic mass of glycopeptides with the signal to noise threshold of more than 3.0 were extracted and transferred to Microsoft Excel. Their intensities of missing glycopeptides were adopted using a Expectation-maximization algorithm^51^. The intensity of each glycopeptide was normalized to the total intensity of the detected subclass glycopeptides. Glycosylation features were calculated as follows:

Agalactosylation = G0F + G0FN in DSIgG2;

Monogalactosylation = G1 + G1F + G1N + G1S + G1FN + G1FS in DSIgG2 or G1 + G1F + G1S + G1FN in DSIgG1;

Digalactosylation = G2 + G2F + G2N + G2FN + G2FS in DSIgG2;

Galactosylation = G1 + G1F + G1N + G1S + G1FN + G1FS +  (G2 + G2F + G2N + G2FN + G2FS)*2 in DSIgG2;

Sialylation = G1S + G1FS + G2FS in DSIgG2;

Fucosylation = G0F + G0FN + G1F + G1FN + G1FS + G2F + G2FN + G2FS in DSIgG2;

Bisecting GlcNAc = G0FN + G1N + G1FN + G2N + G2FN in DSIgG2.

Prior to the variance analysis, PLS-DA was used to test regional diversity of DSIgG glycoforms. Spearman correlation was performed to test the correlation between glycoforms and age and between glycosylation features and age. Mann-Whitney U test was used to find the correlation between glycoforms and sex and the difference in glycoforms between GC patients and BGD patients. And then FDR Controlling Procedures (*i.e.*, Benjamini-Hochberg procedure^52^) were used to obtain the adjusted *p*_*adj*_ values). A *p*_*adj*_ value of less than 0.05 was considered to be statistically significant. ROC analysis was performed to calculate sensitivity and specificity of potential diagnostic variables. Statistical analysis was carried out using SPSS software (version 16.0, SPSS, Chicago, Illinois, USA).

## Additional Information

**How to cite this article**: Zhang, D. *et al.* Disease-specific IgG Fc N-glycosylation as personalized biomarkers to differentiate gastric cancer from benign gastric diseases. *Sci. Rep.* **6**, 25957; doi: 10.1038/srep25957 (2016).

## Supplementary Material

Supplementary Information

## Figures and Tables

**Table 1:** Characteristics of the age-and sex-matched patients with benign gastric diseases (BGDs) and gastric cancer (GC).

Participants	Training set		Validation set	
	BGD	GC	BGD	GC
Age(Mean, range, yr)	52.8, 26–78	53.3, 24–79	56.4, 25–86	56.6, 24–83
Sex(M/F)	30/30	30/30	161/70	161/70

M: male; F: female.

**Table 2:** Comparison of the intensities of glycoforms, glycoform ratios, and glycosylation features between BGD and GC patients.

Glycopeptides	Training Set				Validation Set			
	BGD	GC	*p*value	*p*_*adj*_ value	BGD	GC	*p* value	*p*_*adj*_ value
DSIgG2 G0F	21.80 ± 8.09	24.95 ± 7.77	0.012	0.028	21.85 ± 7.06	23.49 ± 7.64	0.004	0.006
DSIgG2 G1	6.78 ± 2.16	6.78 ± 4.12	0.074	0.113	6.49 ± 2.71	6.25 ± 2.75	0.101	0.144
DSIgG2 G1F	14.79 ± 5.13	15.72 ± 5.43	0.275	0.335	17.65 ± 5.60	18.14 ± 5.43	0.134	0.177
DSIgG2 G2	4.98 ± 1.89	4.15 ± 1.17	0.006	0.017	5.30 ± 1.85	4.99 ± 1.88	0.047	0.074
DSIgG2 G0FN	2.49 ± 1.01	3.19 ± 1.44	0.002	0.005	2.96 ± 1.09	3.31 ± 1.03	<0.001	<0.001
DSIgG2 G1N	1.54±0.75	1.91 ± 1.07	0.034	0.055	1.55 ± 0.75	1.89 ± 1.08	<0.001	0.001
DSIgG2 G1S	3.94 ± 1.58	3.91 ± 2.13	0.332	0.386	3.62 ± 1.60	3.73 ± 1.63	0.487	0.487
DSIgG2 G2F	11.53 ± 3.92	9.24 ± 4.26	<0.001	0.005	10.41 ± 3.65	8.95 ± 3.29	<0.001	<0.001
DSIgG2 G1FN	3.49 ± 1.13	3.26 ± 0.76	0.342	0.389	3.16 ± 0.89	3.03 ± 0.85	0.078	0.118
DSIgG2 G2N	1.98 ± 1.13	2.10 ± 0.92	0.177	0.246	1.84 ± 0.96	1.92 ± 0.96	0.264	0.300
DSIgG2 G1FS	2.57 ± 0.76	2.27 ± 0.68	0.023	0.040	2.20 ± 0.68	2.04 ± 0.64	0.003	0.005
DSIgG2 G2FN	1.60 ± 0.98	2.02 ± 1.05	0.007	0.018	1.35 ± 0.80	1.80 ± 1.06	<0.001	<0.001
DSIgG2 G2FS	3.78 ± 1.91	2.98 ± 1.53	0.013	0.026	3.04 ± 1.47	2.51 ± 1.08	<0.001	<0.001
Agal of DSIgG2	24.29 ± 8.51	28.14 ± 7.89	0.004	0.012	24.81 ± 7.40	26.80 ± 7.88	0.001	0.003
Monogal of DSIgG2	33.12 ± 3.88	33.86 ± 4.10	0.386	0.411	34.69 ± 4.48	35.09 ± 4.29	0.274	0.305
Digal of DSIgG2	23.87±5.25	20.48 ± 5.47	<0.001	0.004	21.94 ± 4.76	20.17 ± 4.48	<0.001	<0.001
Gal of DSIgG2	80.86 ± 11.96	74.82 ± 12.43	0.004	0.013	78.57 ± 11.16	75.44 ± 11.11	0.007	0.011
Sia of DSIgG2	10.30 ± 3.06	9.16 ± 3.40	0.015	0.027	8.86 ± 2.62	8.29 ± 2.67	0.006	0.011
Bi-GlcNAc of DSIgG2	11.10 ± 3.55	12.48 ± 3.51	0.018	0.033	10.87 ± 3.10	11.96 ± 3.41	<0.001	0.001
Fuc of DSIgG2	62.05 ± 7.86	63.63±8.26	0.200	0.264	62.64 ± 9.83	63.28 ± 9.19	0.275	0.299
DSIgG2 G1F/G0F	0.79 ± 0.40	0.72 ± 0.38	0.304	0.361	0.90 ± 0.42	0.86 ± 0.37	0.407	0.415
DSIgG2 G2F/G1F	0.86 ± 0.40	0.63 ± 0.29	<0.001	0.001	0.63 ± 0.27	0.52 ± 0.22	<0.001	<0.001
DSIgG2 G2F/G0F	0.66 ± 0.50	0.44 ± 0.31	<0.001	0.005	0.57 ± 0.48	0.45 ± 0.27	<0.001	<0.001
DSIgG2 G2N/G1N	1.28 ± 0.31	1.29 ± 0.82	0.012	0.027	1.21 ± 0.33	1.08 ± 0.30	<0.001	<0.001
DSIgG2 G1FN/G0FN	1.60 ± 0.79	1.27 ± 0.87	0.002	0.006	1.22 ± 0.62	0.99 ± 0.38	<0.001	<0.001
DSIgG2 G2FN/G1FN	0.45 ± 0.21	0.65 ± 0.38	<0.001	0.002	0.43 ± 0.21	0.62 ± 0.35	<0.001	<0.001
DSIgG2 G2FS/G2F	0.35 ± 0.20	0.35 ± 0.17	0.675	0.675	0.31 ± 0.14	0.31 ± 0.19	0.22	0.262
DSIgG2 G1S/G1	0.72 ± 0.79	0.78 ± 0.58	0.416	0.433	0.69 ± 0.58	0.74 ± 0.51	0.155	0.194
DSIgG2 G0FN/G0F	0.12 ± 0.05	0.15 ± 0.09	0.381	0.414	0.15 ± 0.06	0.16 ± 0.10	0.258	0.300
DSIgG2 G1N/G1	0.23 ± 0.11	0.37 ± 0.29	0.026	0.043	0.27 ± 0.19	0.35 ± 0.28	<0.001	<0.001
DSIgG2 G1FN/G1F	0.28 ± 0.17	0.24 ± 0.11	0.353	0.392	0.21 ± 0.13	0.19 ± 0.11	0.119	0.160
DSIgG2 G2N/G2	0.47 ± 0.36	0.53 ± 0.21	0.014	0.028	0.40 ± 0.40	0.42 ± 0.22	0.003	0.005
DSIgG2 G2FN/G2F	0.16 ± 0.12	0.28 ± 0.24	<0.001	0.001	0.16 ± 0.17	0.25 ± 0.27	<0.001	<0.001
DSIgG2 G1F/G1	2.55 ± 1.50	3.27 ± 2.32	0.089	0.131	3.44 ± 2.34	3.54 ± 1.96	0.092	0.136
DSIgG2 G2F/G2	2.55 ± 1.24	2.50 ± 1.62	0.227	0.291	2.27 ± 1.31	2.06 ± 1.19	0.102	0.142
DSIgG2 G1FN/G1N	2.54 ± 1.02	2.06 ± 1.07	0.013	0.027	2.35 ± 1.04	2.00 ± 1.14	<0.001	<0.001
DSIgG2 G2FN/G2N	0.83 ± 0.29	0.97 ± 0.24	<0.001	0.002	0.77 ± 0.31	0.95 ± 0.25	<0.001	<0.001
DSIgG1 G0F	4.10 ± 2.32	4.41 ± 2.26	0.197	0.266	4.32 ± 2.23	4.67 ± 2.42	0.138	0.177
DSIgG1 G1	1.56 ± 0.84	1.96 ± 1.06	0.006	0.017	1.54 ± 1.12	1.86 ± 1.12	<0.001	<0.001
DSIgG1 G1F	3.97 ± 2.29	3.40 ± 1.77	0.246	0.308	4.45 ± 2.42	4.09 ± 1.87	0.317	0.337
DSIgG1 G1S	3.91 ± 1.30	2.98 ± 1.28	<0.001	0.001	3.33 ± 1.49	2.83 ± 1.11	<0.001	<0.001
DSIgG1 G2F	3.57 ± 1.46	2.77 ± 1.38	0.001	0.005	3.36 ± 1.91	2.65 ± 1.32	<0.001	<0.001
DSIgG1 G1FN	1.61 ± 0.82	2.00 ± 0.88	0.004	0.012	1.57 ± 0.84	1.84 ± 0.95	<0.001	<0.001
Monogal of DSIgG1	11.05 ± 3.20	10.35 ± 3.62	0.153	0.219	10.88 ± 4.19	10.62 ± 3.87	0.394	0.410
DSIgG1 G2F/G1F	1.20 ± 0.85	1.05 ± 1.10	0.038	0.060	0.87 ± 0.67	0.69 ± 0.36	<0.001	<0.001
DSIgG1 G2F/G0F	1.04 ± 0.51	0.70 ± 0.44	<0.001	0.001	0.88 ± 0.48	0.66 ± 0.46	<0.001	<0.001
DSIgG1 G1F/G1	3.48 ± 3.31	2.04 ± 1.22	0.012	0.026	3.96 ± 3.10	2.56 ± 1.25	<0.001	<0.001
DSIgG1 G1S/G1	3.17 ± 1.94	1.74 ± 1.01	<0.001	<0.001	3.11 ± 3.22	1.80 ± 0.94	<0.001	<0.001
DSIgG1 G1FN/G1F	0.73 ± 1.07	0.80 ± 0.88	0.008	0.020	0.52 ± 0.82	0.51 ± 0.28	<0.001	<0.001
DSIgG1 G1FN/G1	1.16 ± 0.59	1.10 ± 0.33	0.611	0.623	1.35 ± 1.20	1.05 ± 0.25	0.212	0.259

Agal: agalactosylation. Monogal: monogalactosylation. Digal: digalactosylation. Gal: galactosylation. Sia: sialylation. Bi-GlcNAc: bisecting GlcNAc. Fuc: fucosylation. G: galactose; F: Fucose; N: N-acetylglucosamine; S: sialic acid. The intensities are shown as mean ± standard deviation.
