# Supplementary material for: Disease-specific IgG Fc N-glycosylation as personalized biomarkers to differentiate gastric cancer from benign gastric diseases
Source: Sci Rep. 2016 May 13;6:25957. doi: 10.1038/srep25957 (PMC4865947; doi:10.1038/srep25957)
Supplement: Supplementary Information [file srep25957-s1.pdf]

Supplementary Information for:

Disease-specific IgG Fc N-glycosylation as personalized biomarkers to differentiate gastric cancer from benign gastric diseases

Dan Zhang[1], Bingchao Chen[2], Yanmin Wang[2], Peng Xia[3], Chengyan He[4], Yujie Liu[1], Ruiqing Zhang[1], Mo Zhang[1], Zhili Li[1]*

[1]Department of Biophysics and Structural Biology, Institute of Basic Medical Sciences, Chinese Academy of Medical Sciences and School of Basic Medicine, Peking Union Medical College, Beijing 100005, P. R. China

[2]Department of Clinical Laboratory, Heze Municipal Hospital, Shandong 274031, P.R. China

[3]Department of Oncology Surgery, First Affiliated Hospital, Xi'an Jiaotong University, Xi'an 710061, P.R. China

[4]Laboratory Medicine Center, China-Japan Union Hospital of Jilin University, Changchun, 130041, P.R. China

***Corresponding author: Zhili Li**, Department of Biophysics and Structural Biology, Institute of Basic Medical Sciences, Chinese Academy of Medical Sciences & School of Basic Medicine, Peking Union Medical College, 5 Dongdan San Tiao, Beijing 100005, P. R. China

E-mail: lizhili@ibms.pumc.edu.cn

Tel/Fax: +86-10-69156479

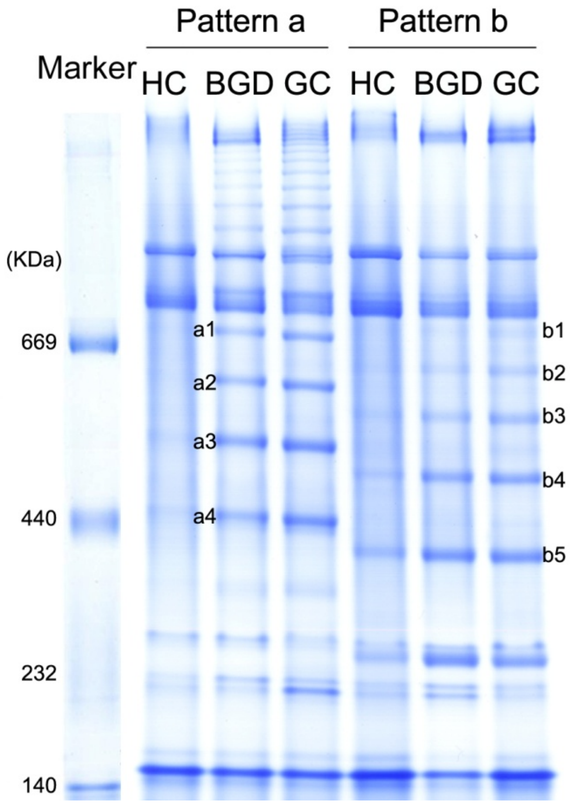

Supplementary Information Fig. S1. Serum IIRPCs isolated by native-PAGE. HC, healthy control. Serum IIRCPs in HC almost disappear.

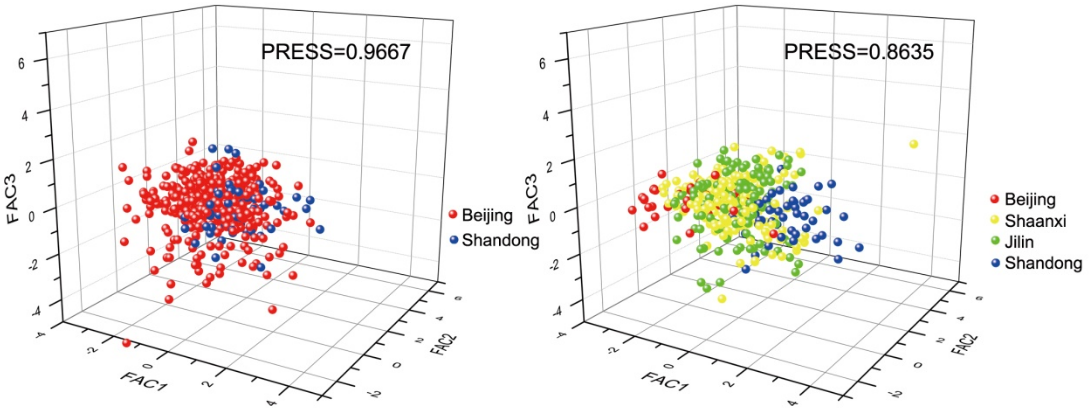

Supplementary information Fig. S2. PLS-DA score of DSIgG Fc glycopeptides detected in patients from different regions.

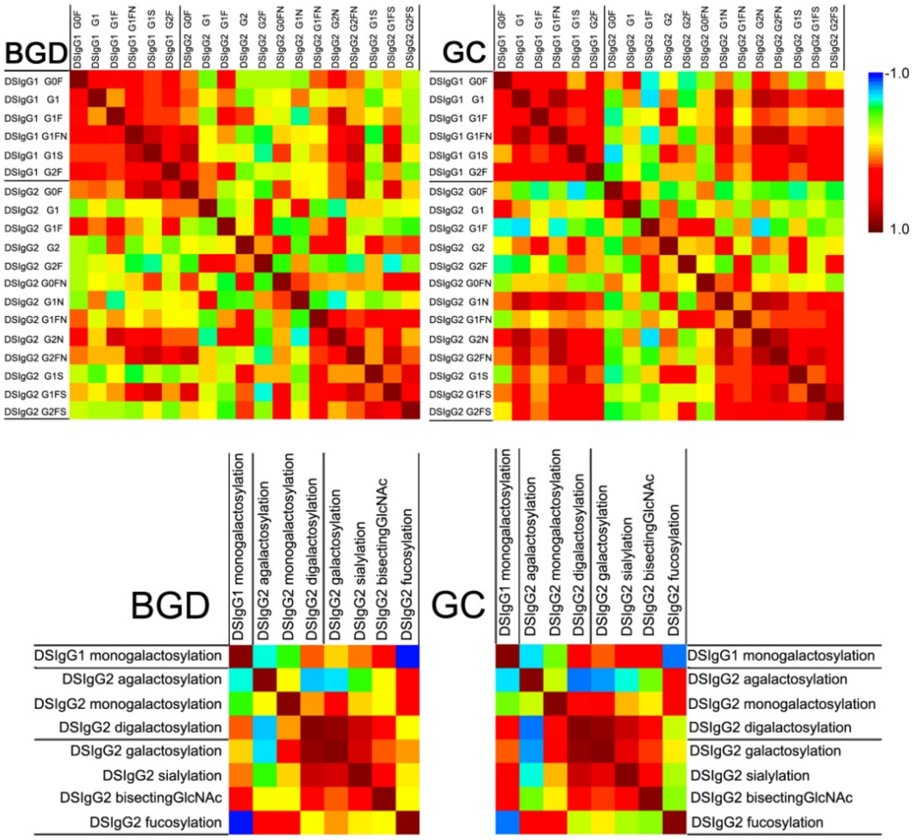

Supplementary Information Fig. S3. Correlation analysis among DSIgG Fc glycoforms and/or its glycoform features in BGD patients or GC patients. Red, positive correlation; blue, negative correlation. -1 to 1 bar indicates the Spearman correlation coefficient.

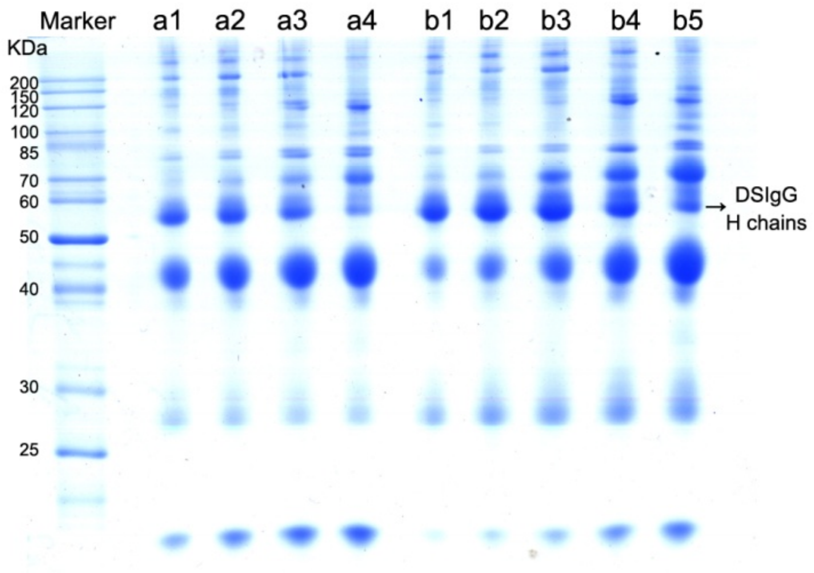

Supplementary Information Fig. S4. DSIgG isolated by SDS-PAGE.

Supplementary Information Table S1. The *m/z* values of the measured glycopeptides of DSIgG1 and DSIgG2 and their corresponding peptide sequences.

Glycoform	IgG1: EEQY**N**STYR $[M+H]^+$		IgG2: EEQF**N**STFR $[M+H]^+$	
	Calculated	Detected	Calculated	Detected
G0F	2634.0459	2634.0484	2602.0561	2602.0565
G0FN	2837.1253	2837.1328	2805.1355	2805.1471
G1	2650.0408	2650.0574	2618.0510	2618.0531
G1F	2796.0987	2796.1062	2764.1089	2764.1141
G1N	2853.1202	2853.1456	2821.1304	2821.1389
G1S	2941.1362	2941.1704	2909.1464	2909.1576
G1FN	2999.1781	2999.1802	2967.1883	2967.1895
G1FS	3087.1941	3087.1898	3055.2043	3055.2046
G2	2821.0936	2821.1389	2780.1038	2780.1091
G2F	2958.1515	2958.1544	2926.1617	2926.1648
G2N	3015.1730	3015.1844	2983.1832	2983.1965
G2S	3103.1890	3103.2292	3071.1992	3071.2266
G2FN	3161.2309	3161.2287	3129.2411	3129.2440
G2FS	3249.2469	3249.2584	3217.2571	3217.2567

Supplementary Information Table S2. Basic characteristics of 846 patients with benign gastric diseases (BGD) and gastric cancer (GC)

Characteristics	Participants	
	BGD	GC
Age(Mean, range, yr)	53.2, 14-90	58.4, 24-83
Sex(M/F)	223/220	302/101
